# MicroRNA-29a Suppresses CD36 to Ameliorate High Fat Diet-Induced Steatohepatitis and Liver Fibrosis in Mice

**DOI:** 10.3390/cells8101298

**Published:** 2019-10-22

**Authors:** Hung-Yu Lin, Feng-Sheng Wang, Ya-Ling Yang, Ying-Hsien Huang

**Affiliations:** 1 Department of Internal Medicine, Kaohsiung Chang Gung Memorial Hospital and Chang Gung University College of Medicine, Kaohsiung 833, Taiwan; linhungyu700218@gmail.com; 2 Mitochondrial Research Unit, Kaohsiung Chang Gung Memorial Hospital and Chang Gung University College of Medicine, Kaohsiung 833, Taiwan; 3 Genomics and Proteomics Core Laboratory, Department of Medical Research, Kaohsiung Chang Gung Memorial Hospital and Chang Gung University College of Medicine, Kaohsiung 833, Taiwan; wangfs@ms33.hinet.net; 4 Department of Anesthesiology, Kaohsiung Chang Gung Memorial Hospital and Chang Gung University College of Medicine, Kaohsiung 833, Taiwan; yaling453@yahoo.com.tw; 5 Department of Pediatrics, Kaohsiung Chang Gung Memorial Hospital and Chang Gung University College of Medicine, Kaohsiung 833, Taiwan

**Keywords:** microRNA-29a, steatohepatitis, fibrosis, CD36

## Abstract

MicroRNA-29 (miR-29) has been shown to play a critical role in reducing inflammation and fibrosis following liver injury. Non-alcoholic fatty liver disease (NAFLD) occurs when fat is deposited (steatosis) in the liver due to causes other than excessive alcohol use and is associated with liver fibrosis. In this study, we asked whether miR-29a could reduce experimental high fat diet (HFD)-induced obesity and liver fibrosis in mice. We performed systematical expression analyses of miR-29a transgenic mice (miR-29aTg mice) and wild-type littermates subjected to HFD-induced NAFLD. The results demonstrated that increased miR-29a not only alleviated HFD-induced body weight gain but also subcutaneous, visceral, and intestinal fat accumulation and hepatocellular steatosis in mice. Furthermore, hepatic tissue in the miR-29aTg mice displayed a weak fibrotic matrix concomitant with low fibrotic collagen1α1 expression within the affected tissues compared to the wild-type (WT) mice fed the HFD diet. Increased miR-29a signaling also resulted in the downregulation of expression of the epithelial mesenchymal transition-executing transcription factor *snail*, mesenchymal markers *vimentin*, and such pro-inflammation markers as *il6* and *mcp1* within the liver tissue. Meanwhile, miR-29aTg-HFD mice exhibited significantly lower levels of peroxisome proliferator-activated receptor γ (PPARγ), mitochondrial transcription factor A TFAM, and mitochondria DNA content in the liver than the WT-HFD mice. An in vitro luciferase reporter assay further confirmed that miR-29a mimic transfection reduced fatty acid translocase CD36 expression in HepG2 cells. Conclusion: Our data provide new insights that miR-29a can improve HDF-induced obesity, hepatocellular steatosis, and fibrosis, as well as highlight the role of miR-29a in regulation of NAFLD.

## 1. Introduction

Non-alcoholic fatty liver disease (NAFLD), which occurs when fat is deposited (steatosis) in the liver due to causes other than excessive alcohol use [1], is among the most common causes of chronic liver disease. With an estimated prevalence ranging from 25% to 45% in most studies, NAFLD increases alongside obesity, diabetes metabolic syndrome, and liver fibrosis [2,3]. Adipose tissue can generate various signals that alter lipid and glucose metabolism, thus leading to hepatic fat accumulation and a proinflammatory milieu that causes cellular injury in the liver and other tissues [1,4]. Increasingly, studies have shown that dietary cholesterol can exacerbate liver fibrosis as free cholesterol accumulates in hepatic stellate cells (HSCs), which increases toll-like receptor 4 signaling and sensitization of HSCs to TGF-β1 [5]. Meanwhile, TGF-β1 may downregulate the peroxisome proliferator-activated receptor γ (PPARγ) expression through β-catenin dependent Wnt signaling pathway, subsequently contributing to increased collagen-1α1 levels in HSCs, potentially resulting in liver fibrosis [6].

The fatty acid translocase protein CD36 facilitates the transport of long-chain fatty acids and can act as an upstream activator of PPARγ to regulate lipid metabolism [7,8]. The liver-specific knockout of CD36 decreases hepatic lipid levels in diet-induced steatosis [9], thus demonstrating its causal role in NAFLD. Fatty acid oxidation (FAO) primarily occurs in mitochondria, as well as in peroxisomes and cytochromes [10,11]. Mitochondrial dysfunction is an important feature of excessive fatty acid influx, while increased FAO produces reactive oxygen species (ROS) and induces oxidative stress in the NAFLD process. This status can further promote damage to the mitochondrial membranes, leading to compromised liver function [12]. Recently, the cytosolic release of mitochondrial components, such as mitochondrial transcription factor A (TFAM) and newly synthesized mtDNA, have been identified as initiators of an innate immune response [13,14], suggesting that mitochondria may play a role in the pathogenesis of nonalcoholic steatohepatitis (NASH).

MicroRNAs (miRNAs) consist of ~22 nucleotide single-stranded non-coding RNAs that can suppress cellular mRNA transcripts and are considered important master regulators of eukaryotic gene expression [15]. Individual mRNAs can be targeted by multiple miRNAs and often have hundreds of mRNA targets [16]. Initial evidence has shown that miR-29 levels are significantly decreased in fibrotic livers, as well as that their downregulation influences HSC activation [17,18,19]. Furthermore, an increase in miR-29 in murine HSCs has been shown to inhibit collagen expression by directly targeting the mRNA expression of extracellular matrix genes [17,18,19]. Recently, our research team has been devoted to exploring the molecular mechanism of miR-29a in the pathogenesis of liver fibrosis. We have already published a series of papers that demonstrate that miR-29a overexpression in cholestatic mice significantly inhibited hepatocellular damage and liver fibrosis, which is involved in various important pathways [20,21,22,23,24,25,26,27]. Furthermore, we have shown that miR-29a improves a methionine-choline-deficient diet, an animal model of non-alcoholic steatohepatitis, induced hepatocellular inflammation, hepatosteatosis, and fibrosis, by targeting DNMT3b expression. This finding highlights the potential of miR-29a targeted therapy for treating non-alcoholic steatohepatitis [28]. Therefore, we used miR-29a transgenic mice (miR-29aTg mice) to determine whether miR-29a could attenuate experimental high fat diet-induced steatohepatitis and liver fibrosis in mice. 

## 2. Materials and Methods

### 2.1. Ethics Statement

Our animal protocol was reviewed and approved by the Institutional Animal Care and Use Committee (IACUC) of Chang Gung Memorial Hospital (#2016121409). We purchased C57BL/6 7-week-old mice from BioLASCO Taiwan Co., Ltd. All animals were housed in an animal facility at 22 °C, with a relative humidity of 55%, in a 12 h light/12 h dark cycle, with food and sterile tap water available ad libitum.

### 2.2. Construction and Breeding of the miR-29a Transgenic Mouse Colony

Transgenic mice with overexpressed miR-29a driven by the phosphoglycerate kinase 1 promoter were bred and housed in a specific pathogen-free rodent barrier, as previously described in another study [1]. Briefly, human phosphoglycerate kinase 1 (PGK) promoter and human miR-29a precursor full-length cDNA were cloned from the cDNA tissue library using polymerase chain reaction (PCR) protocols. A bioinformatic survey indicates that mouse miR-29a (GGAUGACUGAUUUCUUUUGGUGUUCAGAGUCAAUAGAAUUUUCUAGCACCAUCUGAAAUCGGUUAUAAUGAUUGGGGA) presents high homology with human miR-29a (AUGACUGAUUUCUUUUGGUGUUCAGAGUCAAUAUAAUUUUCUAGCACCAUCUGAAAUCGGUUAU) and reveals that putative mRNAs targeted by mouse miR-29a (putative 6228 mRNA targets) are highly similar to human miR-29a (putative 6777 mRNA targets) [22]. The genotype of the transgenic mice was probed with PCR and primers (forward: 5′-GAGGATCCCCTCAAGGAT ACCAAGGGATGAAT-3′ and reverse 5′-CTTCTAGAAGGAGTGTTTCTAGGTATCCGTCA-3′). We obtained the wild-type mice from littermates that did not carry the construct [28]. 

### 2.3. Animal Model and Experimental Protocol

We used five to ten mice for all experiments. The mice were categorized into either the “Chow diet” group or the “high fat (HF) diet” group. In the NAFLD mice model, eight-week-old male C57BL/6J mice (CLEA Japan, Tokyo, Japan) were fed a chow diet or an HF diet (D12331, OPENSOURCE) for 52 weeks. All animals were housed in in a pathogen-free barrier facility accredited by the Association for Assessment and Accreditation of Laboratory Animal Care (AAALAC) at 22 °C, with a relative humidity of 55%, in a 12 h light/12 h dark cycle, with food and sterile tap water available ad libitum. Livers, subcutaneous fat (surrounding the right groin), visceral fat (epididymis fat pads), and intestinal fat (mesenteric fat) were dissected, snap-frozen, and processed to isolate the total RNA and proteins. All specimens were stored at −80 °C until biochemical analysis could be performed.

### 2.4. Open-Field Test

Frequency rearing stand and moving distance were analyzed in the open field. The fear conditioning system (TSE, Bad Homburg, Germany) was used to automatically measure distance travelled (cm) and number of rearing times during the 15-min test. We performed testing in a well-illuminated transparent acrylic cage (30 cm × 30 cm) with a plain black floor, analyzed using multi conditioning system extended advanced 2.0.

### 2.5. Histological Analysis

For morphometric studies, liver tissues were preserved in 10% formaldehyde, embedded in paraffin, and cut into 2-um thick sections stained with hematoxylin-eosin or Masson trichrome stain. The size of the fat droplets was determined through hematoxylin and eosin stain, while liver fibrosis was histologically assessed by quantifying the Masson trichrome stain blue–positive area on 10 low-power (magnification, ×40) fields per slide as previously described in another study [29].

### 2.6. Real-Time RT-PCR

We used the total RNA (2 µg) extracted from liver tissue to generate cDNA with an oligodeoxynucleotide primer (oligo dT15) according to the transcription protocol (Promega, Madison, WI). Then, we followed the manufacturer’s instructions to isolate total microRNA using MicroRNA Isolation Kits (BioChain Institute, Inc, Hayward, CA). Quantitative RT-PCR between both groups were carried out for *col1α1*, *il6, vimentin, snail*, and *mcp1* in the liver. β-actin gene expressions were used to regulate the genes. qPCR was performed in 10 μL 2X SYBR Green PCR Master Mix (Roche) containing 10 mM forward primers and reverse primers and approximately 30 ng cDNA. The relative quantification of gene expression was based on the comparative cycle threshold (CT) method, in which the number of targets was given by 2^−(△CT target−△CT calibrator)^ or 2^−△△CT^. The primer sequences for some of the representative signaling molecules were as follows: *col1α1*: Forward sequence 5’- CTGGCAAGAATGGCGAC-3, Reverse sequence 5’- CCCTGGAGACCAGAGAAG-3’; *il6*: Forward sequence 5′-TTTCCTCTGGTCTTCTGGAGTA-3′, Reverse sequence 5′-CTCTGAAGGACTCTGGCTTTG-3′; *vimentin*: Forward sequence 5′- CACATCGATCTGGACATGCTGT-3′, Reverse sequence 5′- CGGAAAGTGGAATCCTTGCA-3′; *snail*: Forward sequence 5′-GTCTGCACGACCTGTGGAA-3′, Reverse sequence 5′-CAGGAGAATGGCTTCTCACC-3′; *mcp1:* Forward sequence 5′- TTGACCCGTAAATCTGAAGCTA -3′, Reverse sequence 5′- ATTAAGGCATCACAGTCCG -3′; mouse *cd36:* Forward sequence 5′- GCCCAATGGAGCCATCTTTG -3′, Reverse sequence 5′- AGCTGCTACA GCCAGATTCA-3′; human *CD36:* Forward sequence 5′- CTCTTTCCTGCAGCCCAATG -3′, Reverse sequence 5′- CTGCCACAGCCAGATTGAGA -3′; *b-actin* Forward sequence 5′-CAGCCTTCCTTCTTGGGTATG-3′, Reverse sequence 5′- GGCATAGAGGTCTTTACGGATG -3′. For detection of miR-29a expression, predesigned primer/probes for miR-29a (#002112, ThermoFisher) and normalization control sno202 (#001232, ThermoFisher) were used.

### 2.7. Western Blotting

We mixed 30-µg protein extracts with a sample buffer, boiled them for 10 min, and performed electrophoresis using 8–15% sodium dodecyl sulfate-polyacrylamide gels. We transferred the proteins in the gels to a polyvinylidene difluoride membrane and incubated the blots with primary antibodies against COL1α1 (sc-8784, Santa Cruz, CA, USA), PPARγ (16643-1-AP, PROTEINTECH, IL), TFAM (sc-166965, Santa Cruz, CA, USA), CD36/SR-B3 (NB400-144, Novus Biologicals, CO, USA), and glyceraldehyde 3-phosphate dehydrogenase (GAPDH) (60004-1-lg, PROTEINTECH, IL, USA) for protein control. After washing the blots with tris-buffered saline and incubating them with horseradish peroxidase-coupled anti-rabbit immunoglobulin-G antibodies (dilution, 1:5000, NEF812001EA, PerkinElmer, MA, USA), HRP anti-mouse immunoglobulin-G antibodies (dilution, 1:10,000, NEF822001, PerkinElmer, MA, USA),), and HRP anti-goat immunoglobulin-G antibodies (dilution, 1:10,000, sc-2354, Santa Cruz, CA, USA) at room temperature for 1 h, we developed them with enhanced chemiluminescence detection (GE Healthcare Biosciences AB, Uppsala, Sweden), exposed them to film, and quantified the signals by using densitometry.

### 2.8. Luciferase Reporter Assay

With bioinformatics (www.mirbase.org) predicting that miR-29a would target CD36 expression, we hypothesized that miR-29a may also directly affect CD36 mRNA expression in HepG2 cells. The oligonucleotides that contained the mouse CD36 3′ UTR target sequence were annealed and cloned into the pMIR-REPORT™ miRNA Expression Reporter Vector (Applied Biosystems) to generate pMIR-CD36 luciferase plasmid. The sequences in which the miR-29a binding site were replaced with the mutant site were annealed and cloned into the pMIR-REPORT™ reporter vector to generate the pMIR-CD36-Mut luciferase plasmid. We then purified the plasmids using the EasyPrep EndoFree Maxi Plasmid Extraction Kit (BIOTOOLS, Ltd, Taiwan). HepG2 were cultured in 6-cm dishes and transfected with 3 μg of pMIR-CD36 luciferase plasmid or pMIR-CD36-Mut plasmid together with 20 pmol of miR-29a precursor or miRNC (GenePharma). TurboFect reagent (Thermo Fisher Scientific Inc.) was used for transfection, and 48 hours later, luciferase activity was measured using the neolite Reporter Gene Assay System (PerkinElmer).

### 2.9. DNA Isolation and Mitochondrial DNA Copy Number Quantification

Genomic DNA was isolated from snap-frozen livers isolated using the EasyPrep Genomic DNA Extraction Kit (TOOLS), following the manufacturer’s instructions. We quantified the mitochondrial DNA (mtDNA) copy number in 10 μL 2X SYBR Green PCR Master Mix (Roche) containing 5 μM forward primers and reverse primers and approximately 10 ng DNA. The mtDNA levels were assessed using primer against the mitochondrial gene ND1, with telomerase reverse transcriptase (TERT) serving as a loading control. Gene expression quantification was based on the comparative CT method, in which the number of targets was given by 2^−△△CT^. The primer sequences were as follows: *ND1* forward, 5′- ACCAT TTGCA GACGC CATAA-3′; reverse, 5′- TAAAT TGTTT GGGCT ACGG-3′:*TERT* forward, 5′- CTAGC TCATG TGTCA AGACC CTCT-3′; reverse, 5′- GCCAG CACGT TTCTC TCGTT-3′

### 2.10. Statistical Analysis

All values in the figures and tables are expressed as mean ± standard error. Quantitative data were analyzed using one-way analysis of variance when appropriate, and we adopted the least significant difference (LSD) test for post-hoc testing as suitable. Two-sided p-values less than 0.05 were considered statistically significant.

## 3. Results

### 3.1. Overexpression of miR-29a Significantly Reduces Weight Gain, Fat Accumulation in Adipose Tissue, and Liver Weight in the Context of Chronic HFD

**Figure 1:**
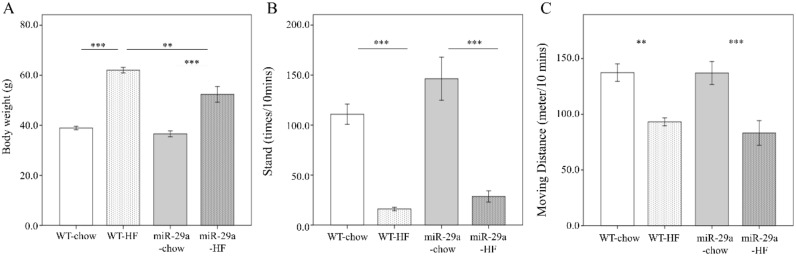
Overexpression of miR-29a reduces weight gain, but has no effect on physical activity in the context of chronic high fat diet (HFD). Weight gain and physical activity of wild type and miR-29aTg mice fed a chow or high-fat diet for 12 months were measured, including (**A**) body weight, (**B**) frequency rearing stand, and (**C**) moving distance documented using a 30 × 30 cm open field box in ten minutes. Data calculated from five to ten mice per group are expressed as mean ± SE. ** *p* < 0.01 and *** *p* < 0.001 between the indicated groups. WT, wild type mice. HFD, high-fat diet. miR-29a, mice harboring overexpression of miR-29a.

**Figure 2:**
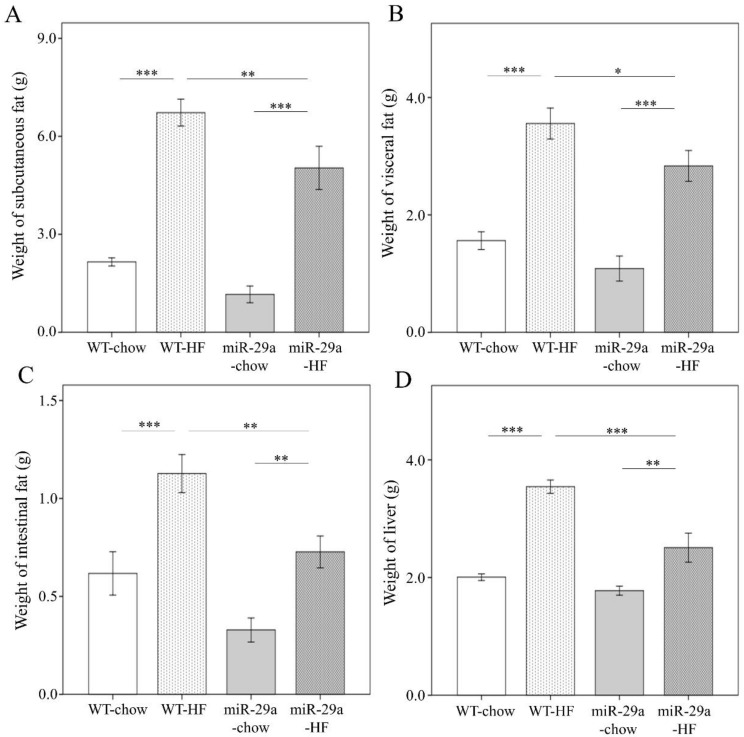
Overexpression of miR-29a significantly reduces fat accumulation in adipose tissue and liver weight in the context of chronic HFD. Various tissue parts were dissected and weighed immediately after sacrifice, with weight of (**A**) subcutaneous, (**B**) visceral, (**C**) intestinal fat tissue, and (**D**) liver. Data calculated from seven to ten mice per group are expressed as mean ± SE. ** *p* < 0.01 and *** *p* < 0.001 between the indicated groups. WT, wild type mice. HFD, high-fat diet. miR-29a, mice harboring overexpression of miR-29a.

To gain insight regarding the potential involvement of miR-29a in the development of NASH, we utilized a high fat diet (HFD) composed of 60% calories from fat to induce an obesity animal model, which has been shown to induce steatosis, hepatic inflammation, and perisinusoidal fibrosis in the liver [30]. The expression level of miR-29a in the liver of WT and miR-29aTg mice was confirmed by qPCR. miR-29aTg mice showed a higher level of miR-29a than WT mice (Figure S1). Both WT and miR-29a mice that were fed HFD showed significant weight gain (both *p* < 0.001, Figure 1A). However, the miR-29aTg-HFD mice had significantly less weight gain compared to WT-HFD mice (*p* < 0.001) (Figure 1A). In both WT and miR-29Tg mice, HFD reduced rearing standing times and locomotion distance in ten minutes (*p* < 0.001, Figure 1B, *p* < 0.01 and *p* < 0.001, respectively, in Figure 1C). Together with weight gain, HFD also induced subcutaneous, visceral, and intestinal fat and liver weight in both WT and miR-29aTg mice (both *p* < 0.001 in Figure 2A, *p* < 0.001 and *p* < 0.01, respectively, in Figure 2B–D). miR-29a-HFD mice presented lower subcutaneous, visceral, and intestinal fat and liver weight than WT-HFD mice (*p* < 0.01, Figure 2A,C, *p* < 0.05 in Figure 2B, and *p* < 0.001 in Figure 2D). This result indicates that an overexpression of miR-29a may reduce fat accumulation and liver mass induced by HFD. 

As shown in Supplementary Figure S2, weight of tissues is normalized by body weight. Despite that weight of all tissues are reduced in miR-29a-HF group compared with WT-HF group (Figure 2), we found that only weight of liver normalized by body weight shows a significant decrease between both groups (*p* < 0.01, Figure S2D). This result reveals that the systemic miR-29a overexpression exerts more prominent impact on proportion of liver weight than fat tissues.

### 3.2. Overexpression of miR-29a Reduces Hepatocellular Steatosis and Fibrosis in the Context of Chronic HFD

**Figure 3:**
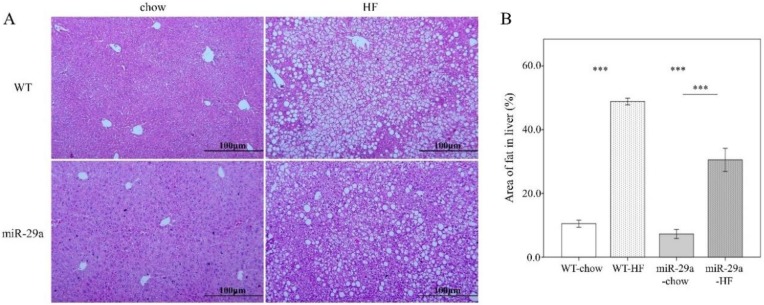
Overexpression of miR-29a reduces hepatocellular steatosis in the context of chronic HFD. Paraformaldehyde-fixed paraffin-embedded liver tissue was used to determine the abundance of lipid droplets with hematoxylin-eosin (HE) stain. (**A**) Representative HE stain image of each group. (**B**) Lipid droplet area quantified using ImageJ. Data collected from three fields of view of each specimen and six to nine specimens for each group are expressed as mean ± SE. ** *p* < 0.01 and *** *p* < 0.001 between the indicated groups. WT, wild type mice. HFD, high-fat diet. miR-29a, mice harboring overexpression of miR-29a.

**Figure 4:**
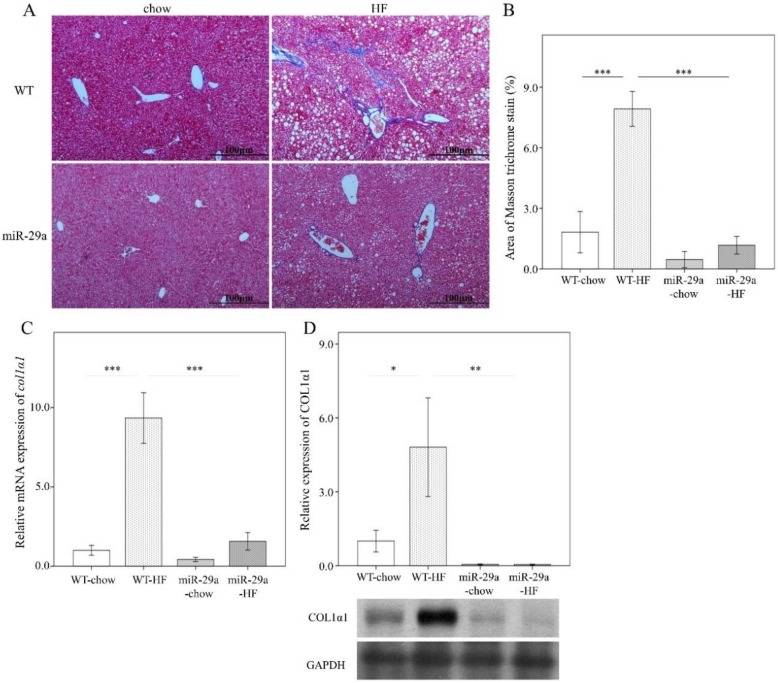
Overexpression of miR-29a reduces liver fibrosis in the context of chronic HFD. Paraformaldehyde-fixed paraffin-embedded liver tissue was used to determine collagen fiber accumulation using Mason’s trichrome stain. Liver tissue stored at −80 ℃ was used for RNA and protein extraction for subsequent qPCR and Western blot experiments, respectively. (**A**) Representative Masson’s trichrome stain image of each group. Blue color indicates positive signal of collagen fiber. (**B**) Positive signal percentage quantified using ImageJ. (**C**) mRNA expression level of *col1α1*, with *β-actin* level as normalization control. (**D**) Representative immunoblotting bands of COL1α1 protein abundance and densitometric results, with glyceraldehyde 3-phosphate dehydrogenase (GAPDH) as the loading control. For the imaging study, data were collected from five fields of view of each specimen and five to eight specimens for each group. For qPCR and Western blot, five to seven specimens were used for each group. Data are expressed as mean ± SE. * *p* < 0.05, ** *p* < 0.01 and *** *p* < 0.001 between the indicated groups. WT, wild type mice. HFD, high-fat diet. miR-29a, mice harboring overexpression of miR-29a.

Next, we examined the hepatic steatosis and fibrotic status in the liver. An abundance of lipid droplets was assayed using hematoxylin and eosin stain. As shown in Figure 3, HFD led to significantly increased fat accumulation in the liver in both WT and miR-29aTg mice (*p* < 0.001 in Figure 3A,B). Of particular note, miR-29aTg-HFD mice expressed a significantly lower droplet count percentage than WT-HFD mice (*p* < 0.001, Figure 3A,B). Liver fibrosis status was determined by collagen fiber accumulation with Masson’s trichrome stain, *col1α1* gene expression with q-PCR, and COL1A1 protein abundance with Western blot. HFD significantly induced the accumulation of collagen fibers in the livers of WT mice (*p* < 0.001 in Figure 4A–C, and *p* < 0.05 in Figure 4D), but not in those of miR-29aTg mice (Figure 4A,B). *COL1A1* gene expression and COL1A1 protein abundance corresponded to the manner of Masson’s trichrome stain (Figure 4C,D). This result indicates that miR-29a overexpression effectively improved hepatocellular steatosis and liver fibrosis induced by HFD.

### 3.3. Overexpression of miR-29a Represses Hepatic Epithelial-to-Mesenchymal Transition (EMT) and Inflammation in the Context of Chronic HFD

**Figure 5:**
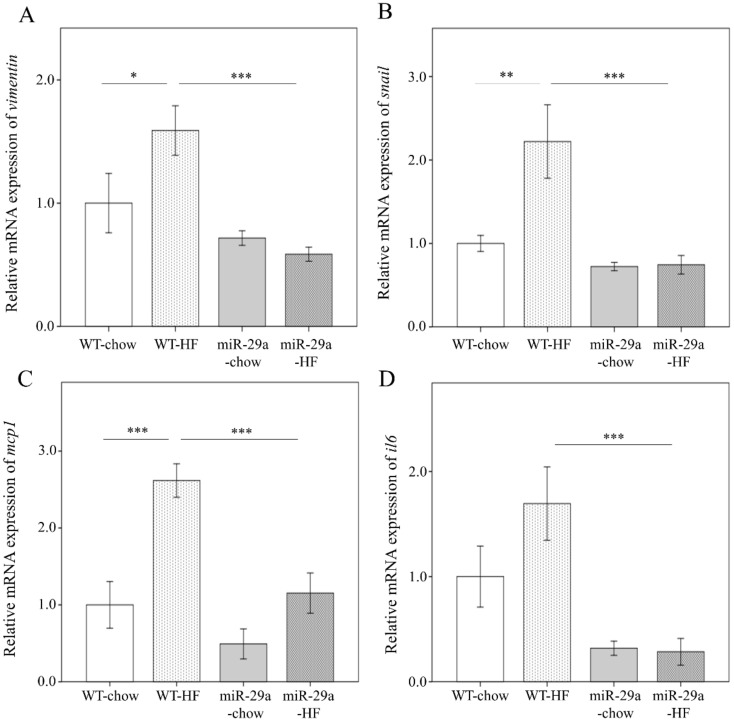
Overexpression of miR-29a represses hepatic epithelial-mesenchymal transition and inflammation in the context of chronic HFD. mRNA expression level of (**A**) vimentin, (**B**) snail, (**C**) mcp1, and (**D**) il6. *β-actin* level is used as the normalization control. Five to seven specimens were used for each group. Data are expressed as mean ± SE. * *p* < 0.05, ** *p* < 0.01 and *** *p* < 0.001 between the indicated groups. WT, wild type mice. HFD, high-fat diet. miR-29a, mice harboring overexpression of miR-29a.

A previous study has reported that the activation of EMT is implicated in liver fibrosis through its response to chronic inflammation [31]. Therefore, we asked whether miR-29a modulates EMT and inflammation in the process of HFD-induced liver fibrosis. The expression level of the EMT-executing transcription factor *snail,* mesenchymal marker *vimentin*, and such pro-inflammation markers as *il6* and *mcp1* were determined using qPCR. As shown in Figure 5, HFD induces a significantly increased expression level of *vimentin* and *mcp1* in WT mice (*p* < 0.05 and *p* < 0.001, respectively), but not in miR-29aTg mice (Figure 5A,C). Notably, miR-29aTg-HFD mice demonstrated a significantly reduced expression of *vimentin*, *snail*, *mcp-1*, and *il6* levels in the liver compared to WT-HFD mice (all *p* < 0.001, Figure 5A–D). This finding suggests that miR-29a overexpression exerts a suppressive effect on EMT and inflammation in the HFD-induced liver fibrosis process.

### 3.4. Overexpression of miR-29a Modulates HFD-Caused Perturbation of Mitochondrial Biogenesis in the Liver

**Figure 6:**
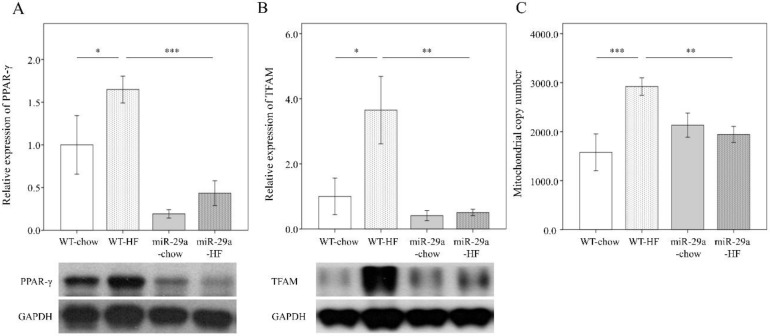
Overexpression of miR-29a modulates HFD-caused perturbation of mitochondrial biogenesis in the liver. Representative immunoblotting bands and densitometric results of (**A**) peroxisome proliferator-activated receptor γ (PPARγ) and (**B**) mitochondrial transcription factor A (TFAM), using GAPDH as the loading control. (**C**) mtDNA copy number per cell probed using qPCR, with *TERT* as the normalization control. Five to ten specimens were used for each group. Data are expressed as mean ± SE. * *p* < 0.05, ** *p* < 0.01, and *** *p* < 0.001 between the indicated groups. WT, wild type mice. HFD, high-fat diet. miR-29a, mice harboring overexpression of miR-29a.

Mitochondrial transcription factor A (TFAM) and mitochondrial DNA (mtDNA) have recently been implicated in initiating the innate immune response [13,32]. PPARγ is a transcriptional activator of upstream TFAM [33], is a regulator in lipid metabolism [34]. Therefore, we looked for a link between mitochondrial biogenesis and inflammation in the progression of liver fibrosis. We measured protein abundance of PPARγ and TFAM with Western blot. MtDNA content was detected using qPCR. As shown in Figure 6, HFD significantly increased the level of PPARγ, TFAM, and mtDNA content in the liver of WT mice (*p* < 0.05, *p* < 0.05, *p* < 0.001 respectively, Figure 6A–C), but not in miR-29aTg mice. miR-29aTg-HFD mice exhibited a significantly lower level of PPARγ, TFAM, and mtDNA content in the liver than WT-HFD mice (*p* < 0.001, *p* < 0.01, *p* < 0.01 respectively, Figure 6A–C). This data reveals that miR-29a overexpression decreases HFD-elicited PPARγ, TFAM, and mtDNA content during the process of liver fibrosis.

### 3.5. miR-29a Inhibits the Expression of Fatty Acid Translocase CD36 by Targeting 3’ UTR

**Figure 7:**
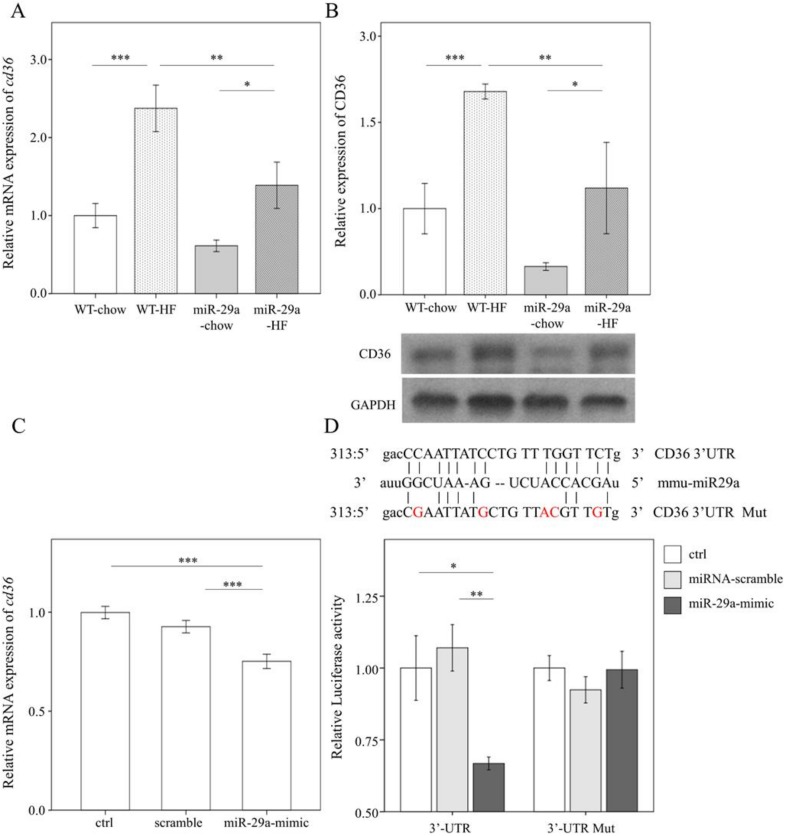
MiR-29a inhibits the expression of fatty acid translocase CD36 by targeting 3’ untranslated region (UTR). (**A**) qPCR analysis of Cd36 in live tissue. (**B**) Representative immunoblotting bands and densitometric results of CD36 in liver tissue. (**C**) qPCR analysis of *cd36* expression of HepG2 cells in vitro after 48h transfection of scramble sequence or miR-29a-mimic. Data obtained from six independent experiments. (**D**) Upper panel, sequence information, and mutual pairing status of CD36-3’UTR, mmu-miR29a, and CD36-3’UTR Mut. Note that red characters represent mismatching sites. HepG2 was first transfected with CD36-3’UTR or CD36-3’UTR mutant luciferase reporter construct then treated with control medium (ctrl), miRNA-scramble, or miR-29a mimic, and finally lysed to detect the luciferase signal. Data are expressed as mean ± SE. * *p* < 0.05, ** *p* < 0.01, and *** *p* < 0.001 between the indicated groups. WT, wild type mice. HFD, high-fat diet. miR-29a, mice harboring overexpression of miR-29a. mmu-miR29a, mouse-origin miR-29a. ctrl, control. Mut, mutant. UTR, untranslated region.

With bioinformatics (www.mirbase.org) predicting that miR-29a will target CD36 expression, we hypothesized that miR-29a may directly affect CD36 expression in hepatocytes. First, we assayed the *cd36* mRNA level and CD36 protein abundance using qPCR and Western blot, respectively. HFD significantly stimulated both mRNA and protein level of CD36 in the livers of both the WT mice and miR-29a mice (*p* < 0.001 and *p* < 0.05, respectively in Figure 7A,B). However, miR-29aTg-HFD mice showed lower both mRNA and protein levels of CD36 than WT-HFD (both *p* < 0.01 in Figure 7A,B). Next, we sought to verify this mechanism by transfecting the miR-29a mimic in vitro in the presence of CD36-3’UTR luciferase reporter construct into human liver hepatocellular carcinoma HepG2 cells. qPCR analysis confirmed that transfection of miR-29a mimic significantly increased expression level of miR-29a in cultured cells (*p* < 0.001, Figure S3). The effect of miR-29a mimic transfection in suppressing *cd36* expression in vitro was also verified (*p* < 0.001, Figure 7C). The mutant form (CD36-3’UTR Mut) of the reporter construct consists of five mismatching sites in contrast to the wild-type form (CD36-3’UTR). This design enables ineffective targeting of mir-29a to CD36-3’UTR Mut compared to normal CD36-3’UTR (upper panel of Figure 7D). The miR-29a mimic significantly reduced luciferase activity in HepG2 cells harboring normal CD36-3’UTR but failed to disturb those harboring the CD36-3’UTR Mut. This result confirms that miR-29a suppresses CD36 expression by targeting its 3’UTR.

## 4. Discussion

NAFLD can progress from simple steatosis to NASH, which can ultimately lead to advanced-stage liver fibrosis and hepatocellular carcinoma [35]. In this study, we revealed the regulatory role of miR-29a in mitigating NAFLD and liver fibrosis, as well as in reducing fat accumulation in liver and adipose tissue in the context of long-term HFD in an animal model. Furthermore, miR-29a overexpression under HFD exerts a repressive effect on proinflammatory cytokine IL-6 and MCP-1, as well as CD36 and PPARγ, which are implicated in the regulation of lipid metabolism. miR-29a suppresses HFD-induced mitochondrial biogenesis, which has been shown to increase in adipose tissue of mice that have been fed HFD [36].

The demonstration of long-term (52 weeks) high fat diet-caused NASH in mice is not only currently scarce but also simulate clinical scenario of NAFLD patients with nutrient excess. The observation is that miR-29aTg mice group presenting suppressed NASH/NAFLD could render a novel mechanism elucidating the role of miR-29a in HF diet-induced liver injury. Despite a modest elevation of miR-29a level (approximate 1.5-fold increase) in the liver of miR-29aTg mice compared to WT mice, NAFLD status is strikingly mitigated in miR-29aTg mice. Thus, one limitation of this study is that the use of whole body miR-29a overexpression may not specify its effect in the liver. To gain mechanistic insight, a mouse model harboring liver-specific miR-29a overexpression/inhibition and rescue of hepatic Cd36 is required. Also, addressing whether in vivo knockdown of Cd36 can show a similar phenotype quantitatively or not will be important. The other limitation of this study is that the mitigative effect in NAFLD observed in mice harboring overexpressed miR-29a from birth may not be sufficient to be translated to an effective intervention. As a result, a rescue experiment using exogenous administration of miR-29a after NAFLD formation would determine its therapeutic potential for clinical application to patients with NAFLD. A recent study by Lambrecht et al. has suggested that miR-29a possesses therapeutic potential in the treatment of live fibrosis [37], indicating this study might pave a way to developing novel clinical treatment of NAFLD.

Mitochondrial dysfunction in hepatocytes is a major and common mechanism in the development of NAFLD [38,39]. Mitochondrial biogenesis is the result of an exquisite mitochondria–nuclear communication, leading to increased mitochondrial mass, in addition to mitochondrial oxidative phosphorylation capacities [40]. PPARγ and its coactivator PGC-1α interact to exert the transcriptional regulation of mitochondrial biogenesis [33]. The transcriptional activity of PGC-1α induces the expression of TFAM [41], which then binds to the control region of mtDNA to regulate its transcription and replication. The extracellular release of TFAM alone can enhance inflammatory cytokine secretion in immune cells [42]. In the context of HFD, the increased expression of TFAM and the elevated mtDNA copy number go hand in hand with increased oxidative damage and the pro-inflammatory markers HIF-1α and p-NFkB in adipose tissue [36]. Our previous study showed that the overexpression of miR-29a results in alleviation of DNA oxidative damage and inflammation, concomitantly improving liver fibrosis induced by the methionine-choline-deficient (MCD) diet [28]. Here, our results further demonstrate that the expression of PPARγ, TFAM, and mtDNA content in the liver is strikingly induced by HFD, but that this induction is neutralized through overexpression of miR-29a. 

Fatty acid translocase CD36 acts as a multifunctional membrane protein that facilitates the uptake of long-chain fatty acid (LCFA) [8] and is also an upstream activator of PPARγ with regard to regulating lipid metabolism [7]. Knockdown of CD36 contributes to the improvement of lipid accumulation in the human hepatic cell line HepG2, indicating its role in lipid over-accumulation [43]. Furthermore, inhibition of CD36 by a pharmaceutical antagonist has been shown to significantly decrease diet-induced weight gain and lipid accumulation both in liver and adipose tissue [44]. Therefore, we exploited the bioinformatic database to predict that CD36 transcripts are the target of miR-29a and further demonstrated that 12 months of HFD causes NASH, along with a significant induction of CD36 protein expression. The overexpression of miR-29a reduces protein expression of CD36 in liver tissue in the context of both chow and high-fat diets. Here, we demonstrated that mice harboring an overexpression of miR-29a presented suppressed CD36 abundance, as well as reduced body weight and adipose tissue of various parts, in line with the notion of targeting CD36 to mitigate obesity. Despite the lack of biochemical measurement of lipid in both liver and blood, which can clarify lipid dynamics in vivo, we demonstrated that a decline in both *cd36* mRNA and CD36 abundance in miR-29aTg group fed with HF diet is compatible with decreased normalized liver weight and liver lipid accumulation determined by IHC compared to WT group, supporting the notion that blunting liver CD36 may reduce hepatocellular steatosis. The interactions between CD36, TGF-β, and mesenchymal markers, such as vimentin and fibronectin, modulate the progression of fibrotic diseases [45]. Yang et al. have demonstrated knockdown of CD36 acts to alleviate renal tubule fibrosis through suppressing TGF-β and fibronectin [46]. In contrast, Kawelke et al. showed that conditional deletion of fibronectin in the mouse liver results in more pronounced fibrosis and stellate cell activation and increased TGF-β-mediated signaling in response to dimethylnitrosamine [47]. Our previous results have demonstrated that miR-29a exerts inhibitory actions on TGF-β signaling-mediated renal fibrosis [48] and bile duct ligation-induced liver fibrosis [49]. Herein, our study shows that miR-29a overexpression exerts protective effect in HFD-elicited CD36 expression, EMT, and liver fibrosis. Nevertheless, weight of whole body, fat, and liver and lipid profile could be affected by basal metabolic rate and appetite. Thus, measurements by using indirect calorimetry or calculating food intake might specify the mechanism accounting for miR-29a overexpression in metabolic profiling.

**Figure 8:**
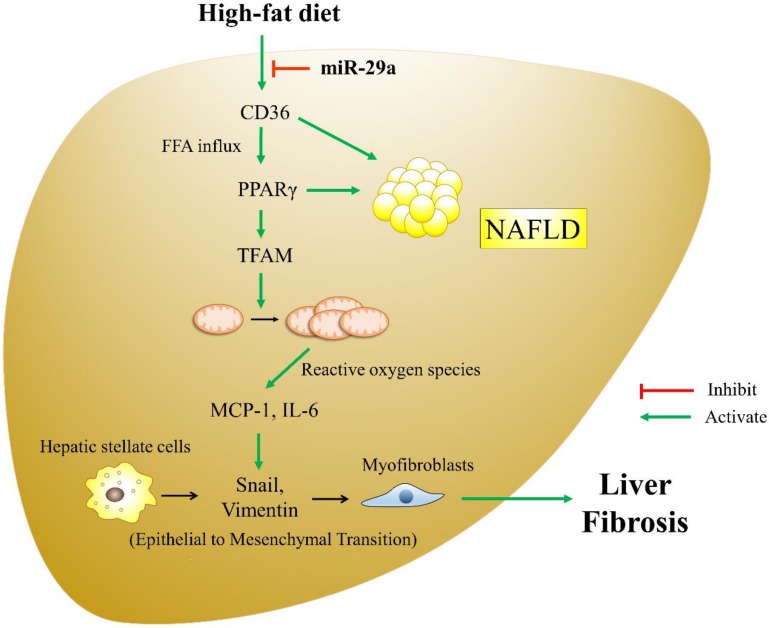
The proposed model of miR-29a exerting a protective effect by targeting CD36 and modulating downstream signaling pathway in HFD-elicited liver fibrosis. HFD causes considerable fatty acid influx into the liver, leading to the up-regulation of PPARγ, TFAM, and mtDNA content. MtDNA and mitochondrial-derived reactive oxygen species can initiate an inflammatory response, leading to the release of such pro-inflammatory cytokines as MCP-1 and IL-6. Chronic inflammation is a stimulator for EMT, which is characterized by the up-regulation of typical makers like snail and vimentin. Activation of EMT facilitates the transformation of hepatic stellate cells to myofibroblasts, contributing to the progression of liver fibrosis. Of particular note, miR-29a can exert an anti-NAFLD effect by targeting CD36 3’UTR and repressing its expression, which may decrease intracellular fatty acid influx, subsequently reducing the up-regulation of PPARγ, TFAM, and mtDNA content, modulating downstream inflammatory response and EMT, and ultimately mitigating liver fibrosis.

During liver fibrogenesis, HSCs are activated and transdifferentiated into contractile myofibroblastic cells that can produce extracellular matrix (ECM) proteins, such as type I collagen [50]. The epithelial-to-mesenchymal transition (EMT) process is characterized by the loss of epithelial cell adhesion molecules and their substitution by such mesenchymal markers as vimentin, fibronectin, collagen, and matrix metalloproteinases [51]. Emerging evidence has suggested that an activated EMT process contributes to HSC trans-differentiation and liver fibrosis [52,53]. With regard to chronic liver injury, EMT can continue to respond to ongoing inflammation and lead to the expression of mesenchymal markers [31]. As such, HSC undergoes constitutive transformation to myofibroblasts, which persistently induce the deposition of ECM and liver fibrosis. Our results demonstrate that miR-29a overexpression reduces the HFD-stimulated EMT driver snail and the mesenchymal marker vimentin. The inhibition of the EMT process may be due to a mitigated inflammatory milieu caused by miR-29a overexpression. Herein, we extended our study on the role of miR-29a in the development of NAFLD in an HFD mouse model. Our investigation of CD36, PPARγ, and mitochondrial DAMPs provides new insight into the crosstalk between metabolism and inflammation in the progression of overnutrition-caused liver fibrinogenesis. The proposed schematics depicting the effect of miR-29a in counteracting HFD-induced liver fibrosis is presented in Figure 8.

In this study, we conclude that the overexpression of miR-29a under long-term HFD can improve hepatocellular steatosis and liver fibrosis, likely by targeting CD36 transcripts and subsequent events, including the reduction of fatty acid flux, the expression of inflammation, and the progression and fibrogenesis of EMT, thus providing a potential target for modulating NAFLD. Furthermore, we also raise a miR-29a-based molecular approach for reducing obesity. Nevertheless, further investigation is warranted to elaborate on the exact mechanism.

## Supplementary Materials

The following are available online at https://www.mdpi.com/2073-4409/8/10/1298/s1.
