# Supplementary material for: MicroRNA-29a Suppresses CD36 to Ameliorate High Fat Diet-Induced Steatohepatitis and Liver Fibrosis in Mice
Source: Cells. 2019 Oct 22;8(10):1298. doi: 10.3390/cells8101298 (PMC6830328; doi:10.3390/cells8101298)
Supplement: Supplementary file 1 [file cells-08-01298-s001.pdf]

Supplementary Materials

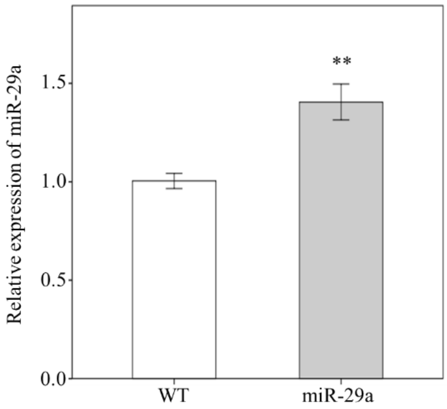

Figure S1. Quantitative results of miR-29a level in the liver. Homogenized liver tissue of WT and miR-29aTg mice was subject to qPCR detection for miR-29a. sno202 used as normalization control. Data calculated from five to six mice per group are expressed as mean ± SE. **p<0.01 compared with WT groups. WT, wild type mice. miR-29a, mice harboring overexpression of miR-29a.

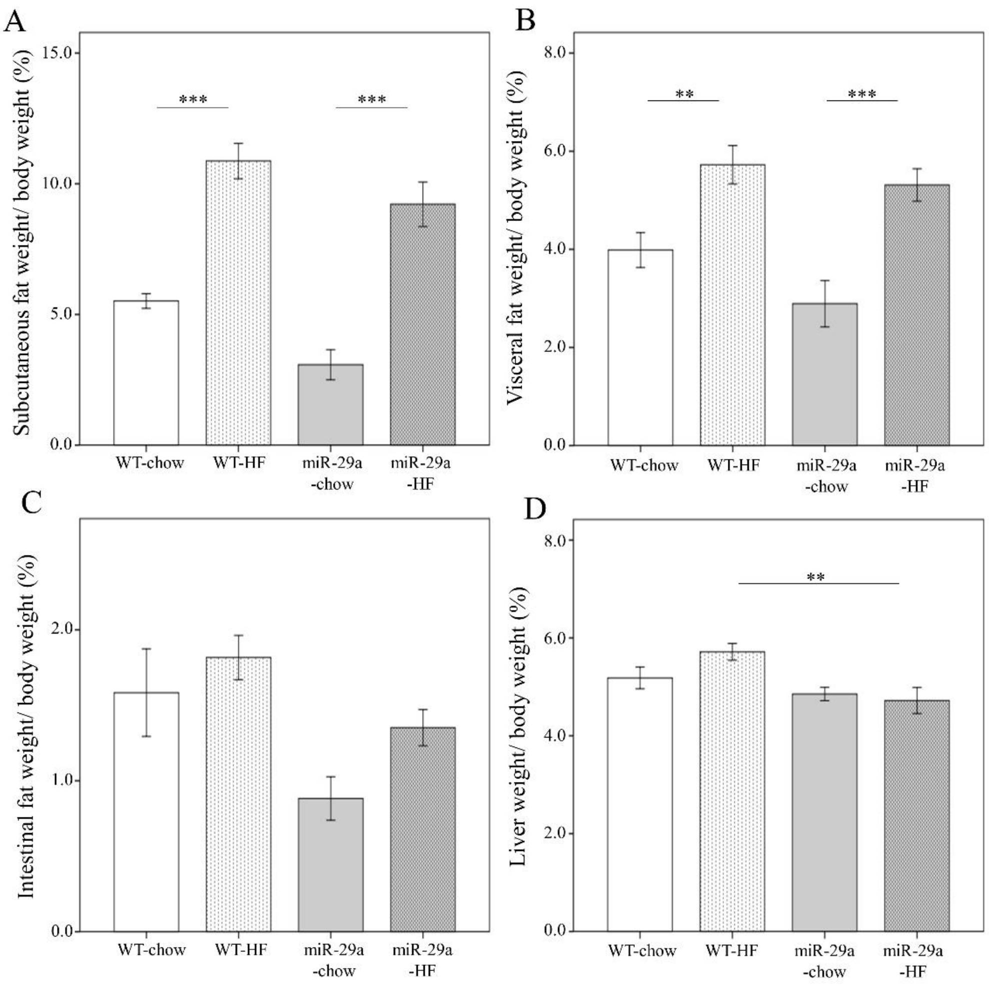

Figure S2. Overexpression of miR-29a exerts most prominent impact on proportion of liver weight than fat tissues. Weight of various tissue (A) subcutaneous, (B) visceral, (C) intestinal fat tissue, and (D) liver were normalized to body weight. Data calculated from seven to ten mice per group are expressed as mean ±SE. **p<0.01 and ***p<0.001 between the indicated groups. WT, wild type mice. HFD, high-fat diet. miR-29a, mice harboring overexpression of miR-29a.

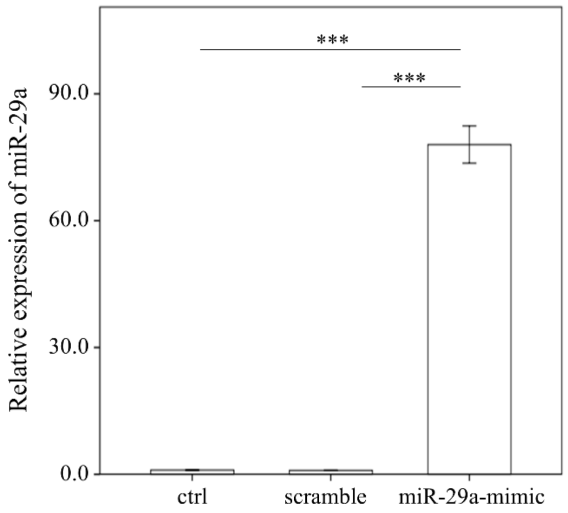

Figure S3. Quantitative results of miR-29a level *in vitro*. qPCR analysis of miR-29a level of HepG2 cells that were transfected with scramble sequence or miR-29-a-mimic for 48 hr. control group (ctrl) treated with equal volume of culture medium. Data calculated from six independent experiments. ***p<0.001 between the indicated groups.
